# An Account of the Remarkable Effects of a Shipwreck on the Mariners; with Experiments and Observations on the Influence of Immersion in Fresh and Salt Water, Hot and Cold, on the Powers of the Living Body

**Published:** 1794

**Authors:** James Currie

**Affiliations:** of Liverpool, M. D. Fellow of the Royal College of Physicians at Edinburgh.


XIII. *An Account of the remarkable Effects of a Shipwreck on the Mariners; with Experiments and Obfervations on the Influence of Immerfion in frefh and falt Water, hot and cold, on the Powers of the living Body. By* James Currie, *of Liverpool, M. D. Fellow of the Royal College of Phyficians at Edinburgh.* Vide *Philofophical Tranfactions of the Royal Society of London, for the Year* 1792. *Part* II. 4to. London, 1792.

THE circumftances of the fhipwreck, which fuggefted the ingenious experiments defcribed in this paper, were as follows:

On the 13th of December, 1790, an American fhip was caft away on a fand-bank that lies in the opening of the river Merfey into the Irifh Channel. The crew got on a part of the wreck, where they paffed the night; and a fignal which they made being difcovered next day from Hillberry ifland, a boat went off, and took up the furvivors. The unfortunate men had remained twenty-three hours on the wreck; and of fourteen, the original number, eleven were ftill alive, all of whom in the end recovered. Of the three that perifhed, one was the mafter of the veffel; another was a paffenger who had been a mafter, but had loft or fold his fhip in America; the third was the cook.

The cook, who was a weakly man, died a few hours before the boat reached the wreck; but the two mafters had been long dead.

Both the mafters, we are told, were ftrong and healthy men, and one of them a native of Scotland, in the flower of life, early inured to cold and hardfhips, and very vigorous both in body and mind. On the other hand, feveral of the furvivors, it is obferved, were by no means ftrong men; and moft of them had been long accuftomed to Carolina and other warm climates; the perfon among the whole who feemed to have fuffered leaft was a negro.

The death of the two mafters was faid to have been owing to their having taken poffeffion of a keg which had contained cherry-brandy, and which ftill contained the cherries; thefe, it was reported, they had kept to themfelves, and eaten in large quantities after the fhipwreck; and this, having produced intoxication, was fuppofed to have haftened their death.

death. Some experienced feamen were fatisfied with this account, which indeed feemed very rational; for though fpirituous liquors may fortify the body againft the effects of heat combined with moifture, and may perhaps fupport it for a fhort time under great fatigue, they would feem, as our author very properly obferves, to be uniformly hurtful when taken under fevere and continued cold. Pleafed to fee a doctrine becoming popular which has been fo ably fupported by Dr. Aikin *, and others, he was induced to think it might receive a ftriking confirmation from this cataftrophe, into the particulars of which he determined to examine accurately. He therefore obtained accefs to the furvivors of the crew, and from them, but more efpecially from Mr. Amyat, the mate, an intelligent young man, he received the information which he required.

From repeated converfations with this perfon, Dr. Currie learnt that Captain Scott, the mafter of the veffel, died in about four hours after the fhip ftruck; and that Captain Davifon, the paffenger, died in about feven: but that

* See Tranfactions of the Philofophical and Literary Society of Manchefter, Vol. I.

the incident of their having eaten the cherries was entirely without foundation: of this Mr. Amyat was certain, for he ſaw the keg which contained them ſtaved, and the cherries, falling on the wreck, were immediately waſhed into the ſea. Mr. Amyat, we are told, expreſſed his ſurpriſe at the early death of the two maſters, but could not aſſign any cauſe for it. He ſaid there was no liquor of any kind ſaved, nor any ſort of food; that the whole crew were on an equality in all points, except that ſome were deeper in the water than others, but that the two maſters had the advantage in this reſpect, for they ſat on the only part of the wreck that was out of the ſea, whereas the negro, who eſcaped almoſt unhurt, was perhaps deepeſt in the ſea of any. Mr. Amyat, it ſeems, explained this in the following manner: when the ſhip ſtruck they cut away her maſts to prevent her from overſetting, and after this ſhe drifted over the ſand-bank, into what he called a "ſwaſh" on the other ſide. Here ſhe floated, and they let go their beſt bower anchor, but it dragged, and the veſſel ſtruck again in a few minutes on another bank. In this ſituation ſhe lay ſome time, beating againſt the ſand, and the ſea breaking over her. In a little while Mr. Amyat ſaw the tar barrels, which which formed her cargo, floating towards the land, and ſoon after the bottom parted entirely, and was carried in the ſame direction. Happily for the men, the part of the wreck on which they were laſhed was held by the anchor, and floated in the water, a ſmall portion of the after part of the quarter deck being above the ſurface. On this ſat the two maſters, generally out of the ſea, but frequently overwhelmed by the ſurge, and at other times expoſed to heavy ſhowers of ſleet and ſnow, and to a high and piercing wind. The temperature of the air, Dr. Currie obſerves, as nearly as could be gueſſed, was from 30° to 33° of Fahrenheit, and that of the ſea, from trials in ſimilar circumſtances, from 38° to 40°. Immediately before the two maſters was Mr. Amyat himſelf. As he was ſitting, and the deck ſloped pretty rapidly, he was generally, we are told, up to the middle in the water; and ſome of the others were up to the ſhoulders. They were not, it is obſerved, at any time able to change their poſition, but kept their legs in pretty conſtant motion to counteract the cold, their arms being employed in holding by the wreck.

The maſter of the ſhip, Captain Scott, a native of North Carolina, and about forty years of age, died firft. As they were in the dark, Mr. Amyat could not fee his countenance; but he was firft alarmed by hearing him talk incoherently, like one in the delirium of fever. By degrees his voice dwindled into a mutter, and his hearing feemed to fail. At length he raifed himfelf up in a fort of convulfive motion, in which he continued a few feconds, and then fell back dead on the deck. This happened about eight in the evening; four hours after the fhip went aground. Soon after this, Captain Davifon, who was about twenty-eight, began to talk incoherently, in the fame manner as the other. He ftruggled longer, but died in the fame way, at about eleven at night. The cook died in the forenoon of the fucceeding day. He was a low-fpirited man, we are told, and defponded from the beginning. All the reft held out, as has been already mentioned, till they were taken up about three in the afternoon. Mr. Amyat faid that his hands and feet were fwelled and numb, though not abfolutely fenfelefs; he felt a tightnefs at the pit of his ftomach, and his mouth and lips were parched; but what diftreffed him moft was cramps in the mufcles of his fides and hips, which were drawn into knots. Though immerfed in the fea, they were were all of them, it ſeems, very thirſty; and though expoſed to ſuch ſevere cold, Mr. Amyat himſelf was not drowſy, nor were any of the men drowſy, nor did ſleep precede death in thoſe that periſhed.

Dr. Currie reflecting on theſe curious facts, had no doubt that the death of the two maſters was to be imputed to their peculiar poſition on the wreck. Expoſed to heavy ſhowers of ſleet and ſnow, they might, he thought, ſuffer from being wet with freſh rather than ſalt water; or from being expoſed to the cold of the atmoſphere, probably ſeven or eight degrees greater than that of the ſea. The chilling effects of evaporation, he conceived, might operate againſt them, promoted as theſe muſt have been by the high wind; or they might receive injury from their frequent immerſions in the ſea, producing an *alternation* in the media ſurrounding. This laſt ſuppoſition, however, did not, he confeſſes, ſtrike him at the time; but the others, he obſerves, dwelt on his mind.

Of the powers attending animation, Dr. Currie remarks, that which ſeems fundamental, is the capacity of the living body of preſerving the ſame heat in various degrees of temperature of the ſame medium, and, indeed, in media of of very different denſity and preſſure. If a definition of life were required, it is, he thinks, on this faculty that it might beſt be founded. It is known, he obſerves, that ſome fluids, applied to the ſkin, vary in their effects according to their impregnation; that in the ſame degree of temperature, for inſtance, pure water on the ſurface of the body is much more hurtful than water in which ſalt is diſſolved. Seafaring men, he remarks, are univerſally acquainted with this, and for a ſtriking proof of the truth, as well as of the importance of the obſervation, he refers us to the Narrative of Lieut. Bligh. Our author thought it probable that the ſaline impregnation might ſtimulate the veſſels of the ſkin, ſo as to counteract the ſedative or debilitating action of the cold. At any rate, it ſeemed to him not unlikely that ſome light might be thrown on this curious ſubject, by obſerving the effects of immerſion in freſh and ſalt water, of equal temperature, on the animal heat; and this, he conceived, might alſo aſſiſt in accounting for the death of the unfortunate men already mentioned. He therefore made the following experiments.

### Experiment I.

A large veffel, containing one hundred and feventy gallons of falt water *, was placed in the open air. The atmofphere was damp and raw. The thermometer, both in the air and in the water, ftood at 44°. The fubject of the experiment was Richard Edwards, a healthy man, twenty-eight years of age, with black hair, and a ruddy complexion. The hour chofen for his immerfion was four in the afternoon, about two hours after his dinner; a time, Dr. Currie tells us, appointed rather for his own convenience, than as being moft proper for the purpofe.

The heat of the perfon who was the fubject of the experiment was 98° before undreffing; his pulfe 100 in the minute. He was undreffed in a room where the mercury was at 56°; and afterwards ftood naked before the fire till his

* In a fubfequent part of his paper the author obferves that the falt water, employed in this and the following experiments, contained falt in the proportion of one to twenty four.

heat and pulſe were examined again, and found as before. He then walked pretty briſkly through a flagged paſſage into an open court, where the north-eaſt wind blew ſharply upon him: he was expoſed to it for a minute, and then plunged ſuddenly into the water up to the ſhoulders. The thermometer, which had been kept in a jug of warm water, at the heat of 100°, was introduced into his mouth, with the bulb under his tongue, as ſoon as the convulſive ſobbings occaſioned by the ſhock were over. The mercury fell rapidly, and a minute and a half after immerſion it ſtood at 87°. He remained motionleſs in the water, and the mercury roſe gradually; at the end of twelve minutes it ſtood at 93°½. While he ſat in the water, it occurred to the author to examine his heat when he roſe out of it into the air. He had reflected, he tells us, on the power that muſt be employed to keep up his heat in a medium ſo denſe as water, and where an inanimate body, of the ſame bulk, would have cooled ſo much more ſpeedily than in air of the ſame temperature. Suppoſing that this heat-producing proceſs, whatever it may be, might continue its operations ſome time after the extraordinary ſtimulus (the preſſure of the water) was removed, moved, he expected to see the mercury rise by the accumulation of his heat, on changing the medium of water for air, and therefore he kept him exposed, naked, to the wind, two minutes after taking him out of the bath. To his surprise, although the attendants were rubbing him dry with towels during this time, the mercury fell rapidly. He was put into a warm bed, and his heat, when examined under the tongue, was 87°, at the axilla 89°. Frictions were used, and brandy mixed with water administered; but Dr. Currie found that the best mode of counteracting the cold, was to apply a bladder, with hot water, to the pit of the stomach, a fact which seems important; this being done, his shiverings, which before were severe, soon ceased, and he became more comfortable. Three hours afterwards, however, he had not entirely recovered his former heat; but by eight at night, he was in all respects as usual.

The author observes that he has been very minute in detailing the circumstances under which this experiment was made; because some of the particulars which, at the time, he thought of little consequence, he found afterwards of importance. The experiment itſelf he determined to repeat as exactly as poſſible.

### Experiment II.

On the next day, at the ſame hour, the ſame perſon was again immerſed as before. His pulſe previouſly was 85, his heat 100°. He had been put to bed an hour before, to ſave the time ſpent in undreſſing. The heat of the water and of the atmoſphere was 44°. The wind was north-eaſt, and ſtrong. On this occaſion, as before, there was a rapid fall of the mercury; the following table ſhows the progreſs of the return of his heat:

| | Ther. | | Ther. |
|---|---|---|---|
| 2 min. after immerſion | 89° ½ | 9 min. after immerſion | 95° ¾ |
| 3 —— - - | 90 ½ | 10 min. - - | 94 ½ |
| 4 —— - - | 92 ½ | 11 —— - - | 95 |
| 5 —— - - | 94 ½ | 12 —— - - | 95 |
| 6 —— - - | 95 | 13 —— - - | 95 ½ |
| 7 —— - - | 95 ¾ | 14 and 15 min. - | 95 |
| 8 —— - - | 95 ¾ | | |

At the end of fifteen minutes he was taken out, and ſtood three minutes, naked, expoſed to the north-eaſt wind, at the end of which time the mercury had ſunk to 88°. A draught of of ale was given him, and he was put into a warm bed; in three minutes after the mercury rose to 93°. An hour after his heat was 95°.

The effects produced by this alternate exposure to water and air of the same temperature, gave a new direction to our author's ideas, and determined him to inquire again into this singular phenomenon. The most obvious method, he observes, would have been to have prolonged the process of alternation, and replunged the person cooled by the external air into the bath; but this, he adds, was running too great a risk, unless some more sudden and certain method could be found of restoring the heat that might be lost. He deemed it prudent, therefore, to proceed more cautiously. In the next experiment he resolved to try the methods of heating as well as cooling the body.

### Experiment III.

On the following day, at the same hour, the same person was again immersed in the salt-water bath. His heat previously was 98°, his pulse 100; the temperature of the water and of the atmofphere, as before, 44°. The mercury funk rapidly to 90°.

| | | | |
|---|---|---|---|
| 2 minutes after the thermometer was at - 88° | | 10 min. after the thermometer was at - | 94°½ |
| 3 min. - - | 88 | 11 min. - - | 94 ¾ |
| 4 —— - - | 88 ½ | 12 —— - - | 95 |
| 5 —— - - | 90 ½ | 13 —— - - | 96 |
| 6 —— - - | 92 | 14 —— - - | 96 |
| 7 —— - - | 92 | 15 —— - - | 96 |
| 8 —— - - | 94 | 16 —— - - | 96 |
| 9 —— - - | 94 | | |

He was now taken out, and ftood in the wind three minutes, fhivering violently. This circumftance, we are told, rendered it difficult to afcertain exactly the fall of the mercury, which was, however, it feems, confiderable. When examined in the room in which he undreffed, it ftood at 90°. He was now plunged into a frefh-water warm bath, heated to 97°½, and yet the mercury fell two degrees.

| | | | |
|---|---|---|---|
| 1 min. after immerfion, in the warm bath, the mercury was at - | 88° | 5 min. after - | 94° |
| 2 min. - - | 92 | 6 —— - - | 96 |
| 3 —— - - | 92 | 7 —— - - | 96 |
| 4 —— - - | 94 | 8 —— - - | 96 |
| | | 9, 10, 11, 12, to 16, | 96 |

Dr. Currie obferves, that if the rife of heat in the cold bath at 44°, and in the warm bath at 97°½, be compared, the firft will be found more more flow ; but that after being fixteen minutes in the one and in the other, the heat was the fame in both cafes, when taken at the mouth. It muft, however, he adds, be acknowledged, that in the cold bath the extremities were chilled and cold, while in the hot bath the heat was equally diffufed.

When the man got out of the hot bath, he put on his clothes, and was remarkably alert and cheerful the whole evening. Encouraged by the fafety of thefe experiments, our author refolved to increafe the time of immerfion in the cold bath, and to inquire more generally into its effects on the fenfations, as well as heat.

### Experiment IV.

At the fame hour of another day, the fame perfon was again immerfed as before, his heat previoufly being 97°½, and that of the water 42° ; the wind was north-eaft, and brifk.

| | | | |
|---|---|---|---|
| 1 minute after, heat | 90° | 12 minutes - - | |
| 2 minutes - - | 92 | 13 ——— - - | |
| 3 ——— - - | 92 | 14 ——— - - | 94°½ |
| 4 ——— - - | 92 ¼ | 15 to 24 - - | 94 ½ |
| 5 ——— - - | 92 | 25 ——— - - | 94 |
| 6 ——— - - | 92 ½ | 26, 27 - - | |
| 7 ——— - - | 94 | 28 ——— - - | 94 ½ |
| 8, 9, 10, 11 - | 94 | 29, 30 - - | 94 |

It will be obferved, that in the above table there are blanks left in the report. At fuch times the thermometer, we are told, was taken out of Edwards's mouth, to admit of his anfwering the queftions put to him. He faid, that on plunging into the water he felt an extreme cold, which he could not but think was partly owing to his being expofed, naked, to the wind before; that this cold diminifhed, and in a little while he felt comfortable, but that after a while the fenfe of coldnefs returned, though lefs than at firft; diminifhing again, but in a lefs degree. At length his fenfations became pretty fixed. In this ftate, when the water was at reft, he fhould not even have known, by his feelings from the upper part of his cheft to the pubes, that he was in water at all. His feet and legs were very cold: fo were his hands and arms; and fo alfo the penis and fcrotum. He mentioned, likewife, that he felt a cold circle round the upper part of his body, though not conftantly. On examining into this, Dr. Currie found it was greateft at firft, and that it extended over the fpace which, from the undulations left in the bath by the plunge of immerfion, was alternately above and under the furface of the water: when the bath fettled, it was little felt; but by agitating the fluid, he could reproduce it, at any time when the cold in the extremities was not ſo great as to prevent its being felt. This curious particular ſerves, our author thinks, to explain a circumſtance much dwelt on by Mr. Amyat, in giving an account of his ſufferings on the wreck; that what he felt moſt ſeverely was the cramps in the muſcles of his hips and ſides, parts which, from his ſituation on the wreck, muſt have been alternately under and above the ſurge. From Mr. Amyat's account, it appeared that the ſea did not break over the ſufferers all the time they were on the wreck. The wind moderated, as well as the waves, and for the laſt fifteen hours they were not at any time overwhelmed, or at leaſt Mr. Amyat himſelf was not. The cold never abated. Being all laſhed to the wreck, they never changed their poſitions: the bodies of thoſe who died occupied the ſpace where they were originally placed. Mr. Amyat, therefore, during the whole time ſat nearly up to the middle in water, but ſubject to the variations occaſioned by the motion of the ſea.

To return to the ſubject of the experiment. When he was expoſed, naked, to the wind, the mercury ſunk as uſual five or ſix degrees, and his ſhiverings were great. With a view to reſtore his heat as ſpeedily as poſſible, the bath was heated to 104° : but after being half a minute in it, he ſcreamed out with pain, eſpecially in his extremities, and about his ſcrotum. When taken out, his ſhiverings we are told, almoſt amounted to convulſion. The bath was lowered to 88°, and he was replaced in it, and its temperature progreſſively, but pretty rapidly, increaſed to 100°. He continued, however, to ſhiver much, his heat remaining about 90° ; but a bladder, with very hot water, being introduced under the ſurface of the bath, and applied cloſe to the ſtomach, the good effects, it is remarked, were inſtantaneous, his ſhiverings ceaſed, and his heat mounted rapidly to 98°.

All theſe experiments having been made on one perſon, Dr. Currie determined to repeat this laſt on another.

### Experiment V.

Rich. Sutton, aged 19, of a pale complexion, and a feebler frame, was immerſed in the bath, under under the circumſtances of the preceding experiment. His heat was previouſly 96°$\frac{1}{2}$.

| | |
|---|---|
| $\frac{1}{2}$ a minute after, heat | 92° |
| 1 minute - - | 90 |
| 2 ——— - - | 88 $\frac{1}{2}$ |
| 3 ——— - - | 89 |
| 4 ——— - - | 90 |
| 5 ——— - - | 92 |
| 6 ——— - - | 92 $\frac{1}{4}$ |
| 7 to 10 - - | 92 |
| 11 ——— - - | |
| 12 to 15 - - | 92 |
| 16 ——— - - | 92 $\frac{1}{2}$ |
| 17 ——— - - | 93 |
| 18 minutes - - | 93°$\frac{1}{4}$ |
| 19 ——— - - | 93 $\frac{1}{2}$ |
| 20, 21 - - | 94 |
| 22 ——— - - | 92 $\frac{1}{2}$ |
| 23 ——— - - | 92 $\frac{1}{4}$ |
| 24 ——— - - | 92 $\frac{1}{4}$ |
| 25 ——— - - | 94 |
| 26 ——— - - | 94 |
| 27 ——— - - | 92 $\frac{1}{2}$ |
| 28 ——— - - | 92 $\frac{3}{4}$ |
| 29 ——— - - | 94 |
| 30 ——— - - | 94 |

Dr. Currie obſerves, that although this perſon ſeemed to bear the cold bath well, having loſt in thirty minutes only 2$\frac{1}{2}$ degrees of heat, yet that when expoſed afterwards to the wind, he ſhivered violently, and loſt his heat very faſt. He was put into a warm bath, heated to 96°, but recovered his heat very ſlowly, as appears from the following table :

| | | |
|---|---|---|
| 1 minute after, heat | 88° | |
| 2 minutes - - | 90 | |
| 2 ——— - - | 90 $\frac{1}{2}$ | |
| 4 ——— - - | 90 | great ſhivering. |
| 5 ——— - - | 90 | here the bath was heated to 100°. |
| 6 ——— - - | 90 | ſhiverings ſtill. |
| 7 ——— - - | 90 | ditto. |
| 8, 9 - - | 90 $\frac{1}{2}$ | ditto. |
| 10 ——— - - | 92 | ditto. |

11 mi-

| | | |
|---|---|---|
| 11 minutes after, heat | 92° | bath heated to 104°. |
| 12 ——— - - | 94 | |
| 13 ——— - - | 93 | —— heated to 108°. Shiverings. |
| 14 ——— - - | 93 | a bladder with very hot water applied to the ſtomach. |
| 15 ——— - - | 94 | |
| 16 ——— - - | 96 | very comfortable. |

### Experiment VI.

Richard Edwards, the original ſubject of experiment, was again immerſed in the cold bath, of the temperature of 40°, and remained in it three quarters of an hour. His heat, it is remarked, was previouſly 97°; his pulſe 90 in the minute. The mercury fell to 92°, was ſtationary for a few minutes, and then mounted, though, as uſual, with no regularity. In twenty-two minutes it ſtood at 96°; it then began to decline, and in twenty-three minutes more had ſunk to 94°. Upon his being expoſed as uſual to the wind, the mercury, we are told, ſunk as before, and he ſhivered violently. In the warm bath at 96° his ſhiverings continued ſeveral minutes, his heat remaining at 90 and 91°. In ſeven minutes the mercury began to riſe faſt, and five minutes after was at 96°.

Expe-

### Experiment VII.

The effects of forty-five minutes immerſion in the cold ſalt-water bath, at 40°, were propoſed to be tried on Richard Sutton. He was, it ſeems, much under the impreſſions of fear, and his heat previouſly raiſed the mercury only to 94°. The mercury, we are told, ſunk, as before, on his immerſion, but to an unuſual degree. It did not ſtop in its fall till it got to 83°, which the author thinks might be in part accounted for by the extraordinary chattering of his teeth, admitting ſome contact of the air. It then mounted in the uſual irregular way, and at the end of thirteen minutes had got to 92°. Here it ſtood for nineteen minutes longer with little variation; at the end of this time it began to fall rapidly, though irregularly, and in three minutes was down at 85°. He had now been thirty-five minutes in the water, and Dr. Currie did not think it ſafe to detain him longer; he therefore hurried him into a warm bath, heated to 96°, where he ſhivered much. The bath was heated gradually to 109°, and in this heat he recovered his proper per temperature in about twenty-eight minutes. Being then put into a warm bed, he fell into a profufe perfpiration, which left him in his ufual health.

With refpect to the ftate of the pulfe in thefe experiments, Dr. Currie obferves that it was not poffible to keep the fubjects of them from fome degree of previous agitation, and that this always quickened the pulfe. The natural pulfe of Edwards, it feems, was about 70 in the minute; but Dr. Currie found that it was never flower than 85 before immerfion, and generally more. However this might be, it invariably, we are told, funk to 65, or from that to 68, in the water, and became firm, regular, and fmall. After being long in the bath, it could hardly be felt at the wrift, but the heart pulfated with great fteadinefs and due force. In the laft experiment, it feems, when the heat funk rapidly, Sutton faid he felt a coldnefs and faintnefs at the ftomach, which he had not perceived before, and the motion of his heart was then found to be feeble and languid. In fome other trials of the effects of immerfion in frefh water, (one of which is related in a fubfequent part of the paper) the fame coldnefs at the ftomach is faid to have preceded a rapid fall of the mercury; and and thefe facts, together with the effects found from applying a confiderable heat to this part when the body was chilled with cold, have convinced our author that there is fome peculiar connexion of the ftomach, or of the diaphragm, or both, with the procefs of animal heat. Whoever, he remarks, will confider the rapidity with which a dead body would have cooled immerfed in water of the temperature of 40°, may form fome eftimate of the force with which the procefs of animal heat muft have acted in the experiments already recited. Thefe experiments, however, he contends, furnifh irrefragable proofs of the futility of fome of the theories of animal heat. The increafe of heat, in fever, he obferves, has led fome perfons to believe that animal heat is produced by, or immediately connected with, the action of the heart and arteries; but in thefe experiments, he remarks, although heat muft have been generated in the bath with more than fourfold its ufual rapidity, the vibrations of the arterial fyftem were unufually flow. Another, and a very beautiful theory of animal heat, continues the author, fuppofes it immediately to depend on refpiration; but in the bath, after the firft irregular action of the diaphragm from the

ſhock of immerſion was over, the breathing, he obſerves, became regular, and unuſually ſlow. Laſtly, the curious phænomenon of the heat riſing and falling, and riſing again, in the bath, with the body at reſt, and the temperature of the ſurrounding medium unchanged, is, he thinks, fatal to thoſe theories of animation which conſider the living body as a mere machine, acted on by external powers, but not itſelf originating action, and differing from other machines only in the peculiarity of the powers which are fitted to ſet it in motion. He has ſaid that the temperature of the medium continued unchanged, but it may be ſuppoſed that the bath was heated a little during the experiments; he allows that it was ſo; but being expoſed, with a large ſurface, to the open air, the wind blowing briſkly over it, its heat, he obſerves, was little altered; in twelve minutes immerſion it had gained nearly one degree, and in forty-five minutes, the longeſt duration of any of the experiments, it had gained three degrees. As this acceſſion was regular, it would not, he obſerves, have invalidated the foregoing obſervations, even if it had been greater.

Many other trials were made on the effects of immerſion in water on the human heat, which the the author ſpeaks of generally, under the general concluſions which they ſuggeſted.

The experiments already recited, ſuggeſted to him the notion, that in all changes from one medium to another of different denſity, though of the ſame temperature, there is a loſs of animal heat. He found, however, that this concluſion requires many reſtrictions.

1. His experiments being made on bodies of ſuch very different denſity as air and water, do not, he obſerves, admit an univerſal inference of this ſort.

2. Being all made in a temperature fifty degrees under the human heat, no certain concluſion, he thinks, can be drawn as to what might happen in degrees of heat much higher, where it is probable the effects of the change, if it appeared at all, might be leſs ſtriking. It would ſeem, however, he obſerves, that after a perſon is long chilled in cold water, the firſt effect of paſſing through the external air into the warm bath, is a fall of heat in the air, and after this a ſtill greater fall in the warm bath, followed, however, by a ſpeedy riſe.

The air and the water being equally cold, and both 45° or under, he found the loſs of heat heat in paffing from the one to the other to be regulated in the following way:

1. If, inftead of being expofed naked to the wind previous to immerfion in the water, the body was kept warm by a flannel covering, the mercury fell much lefs on the firft plunge.

2. If, after plunging into the water, the perfon continued in it only a minute or two, a fubfequent fall of the mercury did not always take place, on his emerging into the air. On the contrary, there was fometimes a rife on fuch occafions of the mercury, efpecially if the atmofphere was at reft.

3. In one inftance, after continuing in the water fifteen minutes, on rifing into the air in a perfect calm, though during a froft, there was little or no feeming diminution of the heat; while expofure under fimilar circumftances, with a north-eaft wind blowing fharply, though the air was many degrees warmer, produced a rapid diminution. The effects of the wind in diminifhing the human heat, are, he obferves, ftriking, and are not, in his opinion, explained by the common fuppofitions.

4. The lofs of heat by a change of media, depends, he thinks, much on the rapidity of the change, for the plaftic power of *life* in varying rying the procefs of animal heat, fo as to accommodate it to the external changes, acts for a time with great celerity, though this celerity feems to diminifh with the ftrength.

### Experiment VIII.

In a large room, where the mercury ftood at 36°, two flipper baths were placed at the diftance of fix yards from each other. One was filled with cold falt water of the temperature of 36°, the other with water heated to 96°, which was the author's own heat. Undreffing himfelf in an adjoining room by a fire, he afterwards flipped on a loofe flannel drefs, and defcended *flowly* into the cold bath, where he remained two minutes; he afcended *flowly* into the air, and then funk himfelf in the warm bath, where he remained two minutes alfo; he returned to the cold bath, where he ftaid two minutes as before, and removed from it again to the warm bath. But during all thefe changes of media and temperature, the thermometer with its bulb under his tongue never varied from 96°. He attributes this partly to the heat of his body being in fome degree defended by the flannel drefs, partly to the calm of the air, but chiefly to the flownefs of motion in thefe changes. He is aware that it may be faid that the time of ftaying in the different baths was not long enough to produce any fenfible change in the heat of circulating fluids of fuch a mafs; but this, he obferves, is not confiftent with many of the other facts.

5. The influence of the application of cold water to the furface of the body on the heat, is in fome refpects, he obferves, regulated by the animal vigour, as the following experiment will fhow.

### Experiment IX.

In the fame room he placed a large empty veffel: in this two young men fat down in fucceffion, each with the bulb of a thermometer under his tongue. A man ftanding on a bench with a bucket containing four gallons of cold falt water, poured the whole of this quantity on the head and fhoulders of each of them, fuffering it to run down on the reft of the body. This procefs took up nearly a minute, during which our author examined the mercury, and found it unchanged. They were were both, we are told, directed to continue fitting without motion for a minute after, during which, in both inftances, the mercury rofe two degrees. A third, much inferior in vigour, fubmitted, it feems, to the fame experiment, and the mercury continued during the affufion of the water unchanged, but in a minute after funk half a degree. In fevers, Dr. Currie obferves, where the heat is generally increafed from two to fix degrees above the ftandard of health, pouring a bucket of cold water on the head always reduces the pulfe in frequency, and commonly lowers the heat from two to four or five degrees. Of this falutary practice he hopes foon to fpeak at large to the public.

6. The power of the body in preferving its heat under the impreffions of cold, and the changes of temperature and of media, feems in fome meafure, our author thinks, to be regulated by the condition of the mind. That fear increafes the influence of cold, and of many other noxious powers, will not, he obferves, be doubted; but the ftate of the mind to which he alludes, is that of *vigorous attention* to other objects. This, it is well known, will, to a certain degree, deaden, or, indeed, prevent, the fenfation of cold; and what does this, he apprehends, prevents, or at leaft weakens, its phyfical action. Thus, in fome fpecies of madnefs, he obferves, where the ideas of imagination are too vivid to admit the impreffions of fenfe, cold is refifted to an extraordinary degree. He has feen a young woman, once of the greateft delicacy of frame, ftruck with madnefs, lie all night on a cold floor, with hardly the covering that decency requires, when the water was frozen on the table by her, and the milk that fhe was to feed on was a mafs of ice.

7. There are, he thinks, particular conditions of the atmofphere, not perfectly underftood, that feem to have an influence in depriving us more fpeedily of our animal heat, than others where the cold is greater.

In addition to his experiments with falt water, Dr. Currie made fome trials to afcertain the effects of immerfion in frefh water on the animal powers, and particularly on the heat; of thefe he has thought it fufficient to relate the following:

EXPE-

### Experiment X.

In the fame veffel, containing an equal bulk of frefh water, Richard Edwards, the fubject of his firft experiments, was immerfed, at the fame hour of the day. His heat previoufly was 98°, his pulfe beat 92 in the minute; the heat of the air was 41°½, that of the water 40°. The wind was in the weft, fo that in the court where the bath ftood there was a perfect calm. As the author had fome fears of the iffue of this experiment, inftead of expofing him for a minute naked to the wind before immerfion, he was covered with a flannel drefs from the air till the inftant he defcended into the water, into which he was fuffered to fink himfelf flowly, with the bulb of the thermometer under his tongue. The following table exhibits the refult;

| | | | |
|---|---|---|---|
| Immediately on immerfion, heat - | 98° | 14 min. after, heat - | 96°½ |
| 1 minute after - | 97 ½ | 15 ——— - . - | 96 |
| 2 minutes - - | 97 | 16, 17, 18, 19, 20 | 96 |
| 3 ——— - - - | 98 | 25 ——— - - | 95 |
| 4 ——— - - - | 97 ½ | 26 ——— - - | 94 |
| 5 ——— - - | 95 | 27 ——— - - | 93 ½ |
| 6 ——— - - | 96 | 28, 29 - - | 94 |
| 7, 8 - - | 96 | 30 ——— - - | 93 |
| 9 ——— - - | 97 | 31, 32 - - | 94 |
| 10 ——— - - | 97 | 33, 34 - - | 92 ½ |

 He

He now, we are told, got out into the air very ſlowly, and ſtood in it three minutes, the wind *not* blowing on him. He loſt one degree of heat at firſt, which he recovered. He was then put into a warm bath at 90°, which at firſt *he felt* warm, and his feet and hands were painful: but in two minutes he fell into a very violent ſhiver, and his heat fell two degrees. The bath was then heated to 95 and 96°, but ſtill he felt cold. It was heated to 99°; he continued in it five minutes, and his heat was 91°. The heat was gradually raiſed to 106°, when the ſenſe of coldneſs of which he had complained at the pit of the ſtomach gradually went off. Before this Dr. Currie had uſually kept him in the warm bath till his natural heat was nearly recovered; but after being half an hour in the heat of 106°, his own heat was ſtill 93°. He now became ſick and very languid, a cold ſweat covered his face, and his pulſe was very quick and feeble. He was removed into bed, but paſſed a feveriſh night, and next day had wandering pains over his body, with great debility, reſembling the beginning ſtage of a fever. By cordials and reſt this went off.

This experiment, the author obſerves, clearly enough enough confirms the greater danger of being wet with fresh than with salt water; but in itself points out nothing certain besides, except that it is not to be rashly repeated. He means, he tells us, to try some of these experiments to a greater extent on the brute creation, when he has procured thermometers better suited to his views. The thermometers he employed had not a sufficient mobility for very nice experiments, and he is aware that in particular instances this may have misled him, though the general results, which is all that is of importance in such experiments as these, will, he hopes, be found just and true.

Towards the conclusion of his paper he offers the following observations on the subject that led to these experiments.

1. It is, he thinks, already well known among seamen, that where there is only the choice of being wet with salt or with fresh water, it is always safest to prefer the first. In the heavy showers of rain, hail, or snow, by which gales of wind are generally accompanied, the men that must be exposed to them, ought, he observes, like Lieutenant Bligh and his crew, to wring their clothes out of salt water.

2. In all cafes where men are reduced to fuch diftrefs by fhipwreck or otherwife, that they can only choofe between the alternative of keeping the limbs conftantly immerged in the fea, or of expofing them to the air while it rains or fnows, or the fea is at times wafhing over them, it is, he thinks, fafeft to prefer a conftant immerfion; becaufe, in the northern regions, where the cold becomes dangerous to life, the fea is almoft always warmer than the air, as the experiments of Sir Charles Douglas fhow *; and becaufe there is not only a danger from the increafed cold produced by evaporation, but alfo from the lofs of heat by the rapid changes of the furrounding medium, as the foregoing experiments point out.

3. Whether, in high and cold winds without rain or fnow, and where a fituation may be chofen beyond the reach of the waves, it is fafer to continue in the air, or to feek refuge in the fea, muft, he thinks, depend upon feveral circumftances, and cannot perhaps be certainly determined. The motives for choofing the fea will, he is of opinion, be ftronger in proportion

* See Philofophical Tranfactions, Vol. LX. p. 39.

portion as the wind is high and cold, and in proportion as the ſhore is bold.

The foregoing narrative, our author obſerves, ſhows that men may ſurvive twenty-three hours immerſion in the ſea, of the temperature of 38 or 40°, (as great a cold as it almoſt ever poſſeſſes) without food or water, and almoſt without hope of relief; but that any man, he adds, ever ſurvived an equally long expoſure to the higher degrees of cold of the atmoſphere, in the ſame circumſtances, does not appear. Though in the caſe related, immerſion in water did not prevent thirſt, yet there is no doubt, he thinks, that it alleviated it; a circumſtance of high importance, he obſerves, towards the preſervation of life.

In a poſtſcript to his paper, Dr. Currie remarks that he has purpoſely avoided any reaſoning on the cauſes of the loſs of vital heat on the change of media in the experiments recited. It may, he is aware, be ſuppoſed that during immerſion, the water immediately in contact with the ſkin having become heated to a certain degree, the naked body, on riſing from it into the air, was in fact expoſed to a colder medium, and thus the loſs of heat, in this inſtance, produced. His examination of the heat of the water during immerſion not having been made in contact with the body, he does not deny that there is fome foundation for the fuppofition; and the cafes, he allows, are by no means exactly parallel between immerfion in an open veffel, however large, and immerfion in the fea, where the conftant undulation may be prefumed to occafion a continual change in the furrounding fluid. But whatever allowance may be made for the circumftance mentioned, he is perfuaded that the difference between the denfity of air and water being confidered, it is not fufficient to explain the lofs of heat in the inftance alluded to. The changes of temperature in the living body are governed, he obferves, by laws peculiar to itfelf. He has found, in certain difeafes, greater and fuddener variations than any mentioned, from applications of cold very gentle in degree, and momentary in duration.

Mr. Hunter, in his "Experiments and Obfervations on Animals producing Heat," has objected to taking the heat of the human body by introducing the bulb of the thermometer into the mouth, becaufe it may be affected by the cold air in breathing. This objection our author allows to be founded, if the bulb be placed on the upper furface of the tongue; but if it be under it, and the lips fhut, the effects of refpiration, he affures us, may be difregarded, as he has found from many hundred experiments. The heat, we are told, may be obferved in this way with eafe and certainty, by employing thermometers curved at that end to which the bulb is affixed, (the bulb being introduced at the corner of the mouth) fome of which have been made for him by Mr. Ramfden, according to a form given, as well as others on Mr. Hunter's plan. From repeated trials it appears to him, that when the ufual clothing is on, the heat of the living body may be taken, with nearly the fame refult and equal certainty, under the tongue with the lips fhut, at the axilla with the arm clofe to the fide, and in the hollow between the fcrotum and the thigh; but that every other part of the furface is liable to variation and uncertainty. It is evident, he obferves, that of thefe three methods, the firft only can be employed when the trunk of the body is immerfed in water; and even when the naked body is expofed to the cold air, the firft method feems to him the beft, the heat remaining moft fteady under the tongue: the axilla, according to his experience, is the next beft in order; and the worft, the lower part of the groin: for the fcrotum and the parts parts of generation, he obferves, lofe their heat on the application of cold more fpeedily, perhaps, than any other part of the body, the extremities not excepted.

